# Effects of diazepam on hippocampal blood flow in people at clinical high risk for psychosis

**DOI:** 10.1038/s41386-024-01864-9

**Published:** 2024-04-24

**Authors:** Nicholas R. Livingston, Amanda Kiemes, Gabriel A. Devenyi, Samuel Knight, Paulina B. Lukow, Luke A. Jelen, Thomas Reilly, Aikaterini Dima, Maria Antonietta Nettis, Cecilia Casetta, Tyler Agyekum, Fernando Zelaya, Thomas Spencer, Andrea De Micheli, Paolo Fusar-Poli, Anthony A. Grace, Steve C. R. Williams, Philip McGuire, Alice Egerton, M. Mallar Chakravarty, Gemma Modinos

**Affiliations:** 1 Department of Psychological Medicine, Institute of Psychiatry, Psychology, and Neuroscience, King’s College London, London, UK; 2 Department of Psychiatry, McGill University, Montreal, QC Canada; 3 Cerebral Imaging Centre, Douglas Mental Health University Institute, Montreal, QC Canada; 4 Institute of Cognitive Neuroscience, University College London, London, UK; 5 Department of Psychosis Studies, Institute of Psychiatry, Psychology, and Neuroscience, King’s College London, London, UK; 6 Department of Psychiatry, University of Oxford, Oxford, UK; 7 Department of Neuroimaging, Institute of Psychiatry, Psychology, and Neuroscience, King’s College London, London, UK; 8 Outreach and Support in South-London (OASIS) service, South London and Maudsley (SLaM) NHS Foundation Trust, London, UK; 9 Department of Brain and Behavioural Sciences, University of Pavia, Pavia, Italy; 10 Departments of Neuroscience, Psychiatry and Psychology, University of Pittsburgh, Pittsburgh, PA USA; 11 MRC Centre for Neurodevelopmental Disorders, King’s College London, London, UK

**Keywords:** Diagnostic markers, Drug development, Psychosis, Transporters in the nervous system

## Abstract

Elevated hippocampal perfusion has been observed in people at clinical high risk for psychosis (CHR-P). Preclinical evidence suggests that hippocampal hyperactivity is central to the pathophysiology of psychosis, and that peripubertal treatment with diazepam can prevent the development of psychosis-relevant phenotypes. The present experimental medicine study examined whether diazepam can normalize hippocampal perfusion in CHR-P individuals. Using a randomized, double-blind, placebo-controlled, crossover design, 24 CHR-P individuals were assessed with magnetic resonance imaging (MRI) on two occasions, once following a single oral dose of diazepam (5 mg) and once following placebo. Regional cerebral blood flow (rCBF) was measured using 3D pseudo-continuous arterial spin labeling and sampled in native space using participant-specific hippocampus and subfield masks (CA1, subiculum, CA4/dentate gyrus). Twenty-two healthy controls (HC) were scanned using the same MRI acquisition sequence, but without administration of diazepam or placebo. Mixed-design ANCOVAs and linear mixed-effects models were used to examine the effects of group (CHR-P placebo/diazepam vs. HC) and condition (CHR-P diazepam vs. placebo) on rCBF in the hippocampus as a whole and by subfield. Under the placebo condition, CHR-P individuals (mean [±SD] age: 24.1 [±4.8] years, 15 F) showed significantly elevated rCBF compared to HC (mean [±SD] age: 26.5 [±5.1] years, 11 F) in the hippocampus (*F*(1,41) = 24.7, *p*_FDR_ < 0.001) and across its subfields (all *p*_FDR_ < 0.001). Following diazepam, rCBF in the hippocampus (and subfields, all *p*_FDR_ < 0.001) was significantly reduced (*t*(69) = −5.1, *p*_FDR_ < 0.001) and normalized to HC levels (*F*(1,41) = 0.4, *p*_FDR_ = 0.204). In conclusion, diazepam normalized hippocampal hyperperfusion in CHR-P individuals, consistent with evidence implicating medial temporal GABAergic dysfunction in increased vulnerability for psychosis.

## Introduction

Transformations in our understanding of the nature, etiology, and early course of psychotic disorders drove a marked re-orientation of mental health services toward early intervention in individuals at clinical high risk for psychosis (CHR-P), raising the prospect that prevention of psychosis onset may be a realistic goal [1, 2]. Despite this progress, current treatments have a minimal influence on transition to psychosis [3, 4], and there is no robust evidence to favor any single preventive intervention over another [5, 6]. A better understanding of the neurobiology underlying the CHR-P phenotype is critical for the much-needed development of interventions to prevent psychosis onset.

Postmortem, preclinical, genetic, and clinical neuroimaging evidence suggests that hippocampal abnormalities are central to the pathophysiology of psychosis [7–9], thus representing a potential therapeutic target. Hippocampal dysfunction in psychosis is proposed to arise from a disruption in neural excitatory/inhibitory balance, likely driven by GABAergic inhibitory interneuron dysfunction [10–14], although glutamatergic receptor dysfunction is also implicated [15, 16]. This imbalance would render the hippocampus dysrhythmic and hyperactive [17], and excessive glutamatergic output [18–21] from this region to the striatum, amygdala, and prefrontal cortex may underlie the development of positive, negative, and cognitive symptoms, respectively [22]. Such network properties are supported by tractography evidence, demonstrating for example monosynaptic projections from the ventral hippocampus to the striatum [23]. Preclinical findings in the well-validated methylazoxymethanol acetate (MAM) rodent model of neurodevelopmental disruption [24–26] indicate that a loss of hippocampal parvalbumin-expressing (PV+) inhibitory interneurons [27] leads to hippocampal hyperactivity as measured with electrophysiology [28, 29]. Other rodent models have also shown that selective reduction in PV mRNA expression [30] or knock-out of PV+ interneuron [28, 29] or a glutamate-metabolizing enzyme [31] are each sufficient to induce hippocampal hyperactivity. In MAM-treated rats, hippocampal hyperactivity leads to striatal hyperdopaminergia [25], which is a core neurobiological feature of positive symptoms in schizophrenia [22]. This hippocampal hyperactivity can be normalized by hippocampal chemical inactivation [26, 32], or by pharmacologically facilitating GABA signaling through direct hippocampal infusion of either a nonselective (the benzodiazepine midazolam) [33] or selective positive allosteric modulator (PAM) for α5-subunit-containing GABA_A_ receptors [33, 34]. Furthermore, repeated administration of the benzodiazepine diazepam during the peripubertal period to MAM-treated rats prevented the development of psychosis-relevant features in adulthood [35–37], highlighting the prophylactic potential of GABA-enhancing compounds for psychosis.

In CHR-P individuals, hippocampal hyperactivity has been observed in vivo [38–42], which – as a result of neurovascular coupling – is associated with increased regional cerebral blood flow or volume (rCBF or rCBV) by arterial spin labeling (ASL) or gadolinium-contrast magnetic resonance imaging (MRI), respectively. Elevated hippocampal rCBF in CHR-P individuals has been associated with elevated striatal pre-synaptic dopamine synthesis capacity [43] and medial prefrontal cortex GABA concentrations [44]. Baseline elevated hippocampal rCBF or rCBV was also found to predict higher positive and negative symptom severity [38], poor functioning [38, 43], and transition to psychosis [38, 39, 44], and to normalize in those individuals who remit from the CHR-P state [40]. Within the hippocampus, hyperactivity is proposed to originate in the CA1 subfield and then extend to the subiculum and beyond [9, 38, 39, 42]. Hence, hippocampal rCBF may be a marker for symptom severity and psychosis onset in CHR-P individuals, which preclinical evidence suggests may be prevented by pharmacological enhancement of hippocampal GABA levels [35–37].

Mechanistic research focussed on whether a well-characterized, marketed GABA-enhancing compound such as a benzodiazepine can downregulate hippocampal hyperactivity in CHR-P individuals would provide fundamental evidence for the development of more hippocampal-specific GABA-enhancing compounds for the at-risk stage. Prior positron or xenon emission tomography (PET or XET) research demonstrated global [45–48] and temporal lobe [45] reductions in CBF under an acute non-sedating dose of a benzodiazepine in healthy controls. However, no such studies have investigated the hippocampus specifically, or included CHR-P individuals. In the present experimental medicine study, we compared the acute effects of a single dose of diazepam vs. placebo on ASL-derived rCBF in the hippocampus and its subfields in a sample of antipsychotic-naïve CHR-P individuals. To determine baseline alterations in the CHR-P group, rCBF measures from CHR-P individuals under placebo were first compared to those from a sample of healthy controls (HC). We hypothesized that, compared to HC, CHR-P individuals under placebo would show increased rCBF in the hippocampus and its subfields, which would be most apparent in the CA1 subregion [38, 39]. Additionally, based on preclinical evidence [34], we hypothesized that a single dose of diazepam would reduce hippocampal rCBF in CHR-P individuals compared to placebo, and that this would be observed across all subfields due to their similar levels of GABA_A_ receptor expression [49, 50]. For completeness, supplementary analyses examined broader effects of diazepam on voxel-wise grey matter (GM) rCBF and associations between baseline levels of symptoms/functioning and hippocampal rCBF in CHR-P individuals.

## Materials and methods

### Participants

Twenty-four CHR-P individuals, aged 18–32, were recruited from OASIS (Outreach and Support in South London), an early-intervention service within the South London and Maudsley NHS Foundation Trust, UK [51]. CHR-P criteria was determined using the Comprehensive Assessment of At-Risk Mental State (CAARMS) [52]. All individuals were required to be experiencing current attenuated psychotic symptoms, defined as having a severity and frequency score of ≥3 on P1-P4 of the CAARMS. Exclusion criteria included a psychosis/neurological disorder diagnosis, previous/current exposure to antipsychotics, current exposure to psychotropic medications with direct GABAergic/glutamatergic action (except for antidepressants, see Supplementary Methods for list of drug types), pregnancy/breastfeeding, IQ < 70, and any contraindication to MRI scanning. A flowchart of study participation, outlining participant dropouts, is displayed in Supplementary Fig. 1.

To validate hippocampal hyperactivity in this sample of CHR-P individuals, we utilized data from 22 HC from a previous study [53], acquired with the same MRI scanner, scanning sequences, and acquisition parameters as the CHR-P sample (see Supplementary Methods). HC data were reanalyzed with the same preprocessing steps as the CHR-P data. While HC participants were not assessed with the CAARMS, they scored very low on self-report questionnaires of schizotypy (measuring psychotic-like experiences) and were assessed with the Mini International Neuropsychiatric Inventory in order to exclude personal history of neurological/psychiatric disorders. Further details on recruitment and inclusion/exclusion criteria are described in the original publication [53].

### Study design and procedure

This experimental medicine study had full ethical approval from the National Health Service UK Research Ethics Committee and was carried out at King’s College London. While the study received ethical clearance as ‘not a Clinical Trial of an Investigational Medicinal Product’ by the EU directive 2001/20/EC, it was registered on clinicaltrials.gov (NCT06190483). All participants provided written informed consent. Using a randomized, double-blind, placebo-controlled, crossover design, the 24 CHR-P participants underwent two MRI sessions, once under a single oral dose of diazepam (5 mg) and once under oral placebo (50 mg ascorbic acid), with a minimum 3-week washout period between visits. In the assessment visit, demographic information, basic medical history, and clinical/cognitive assessments were collected (CAARMS [52], Global Functioning Social and Role scales [54], Hamilton Anxiety and Depression scales [55, 56], Wechsler Adult Intelligence Scale III [57], Trail Making Test A & B [58]). At each scanning visit, the diazepam/placebo capsule was administered 60 min prior to MRI scanning, so that the MRI session coincided with peak diazepam plasma levels [59]. Further study procedure details can be found in the Supplementary Methods.

### MRI acquisition

MRI scanning for all participants was conducted at the Centre for Neuroimaging Sciences, King’s College London, using a General Electric MR750 3.0 T MR scanner with an 8-channel head coil. rCBF was measured using a 3D pseudo-continuous ASL sequence (multi-shot 3D Fast Spin Echo Stack of Spirals) as used in previous CHR-P studies from our group [40, 41, 53], and T1-weighted SPGR and T2-weighted FRFSE images were also acquired. Further acquisition details can be found in Supplementary Methods.

### Image processing

#### Generation of hippocampal/subfield and total GM masks

**Fig. 1:**
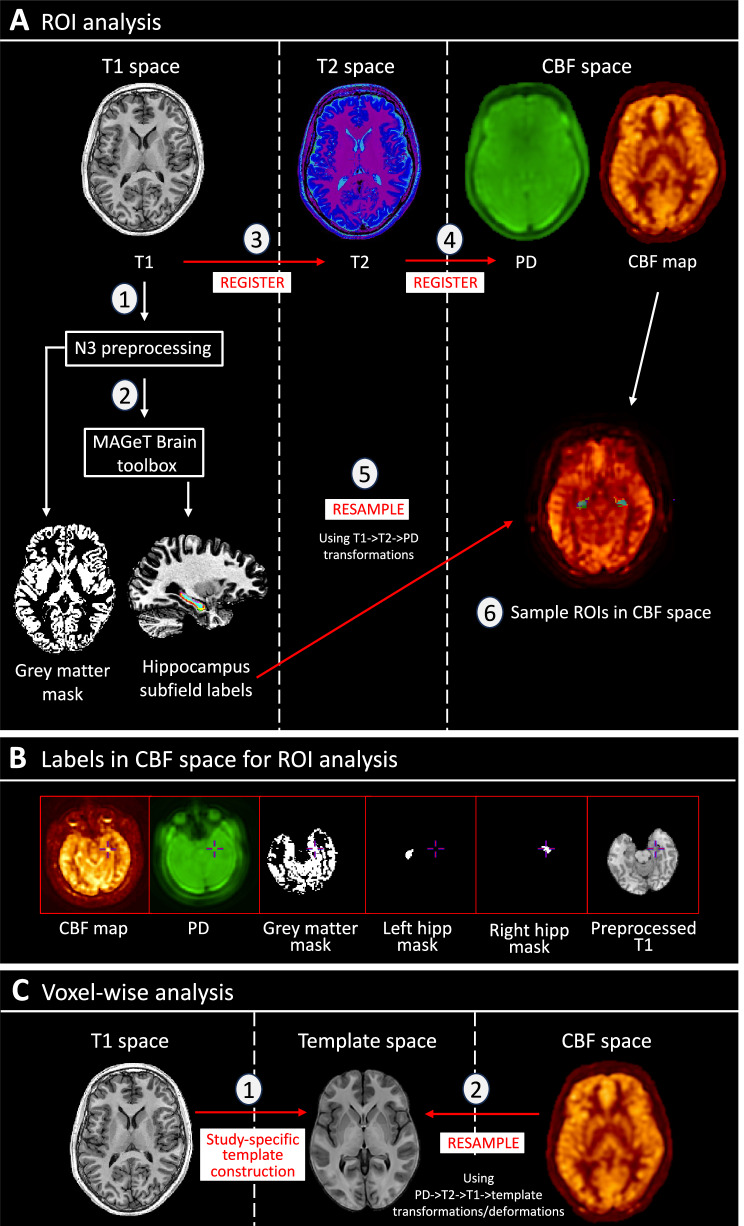
ASL preprocessing and analysis pipeline. **A** Diagram showing pipeline for region-of-interest (ROI) analysis. T1 images were preprocessed (1) and run through the MAGeT Brain toolbox (2), generating masks for grey matter, whole hippocampus, and hippocampal subfields. Using T1- > T2- > PD transformations calculated from registration (3 & 4), these masks were resampled (5) to CBF space (single resampling step) to allow for sampling of rCBF in native space (6). **B** Demonstration of optimum registration between CBF map, masks, and T1 images. **C** Schematic showing steps for voxel-wise analysis for CHR-P diazepam vs. placebo. A study-specific template was generated (1) from participant-averages (averaged structural scans from both drug conditions), and the CBF maps were resampled (2) to common space (single resampling step calculated from PD- > T2- > T1-> template transformations/deformations). hipp hippocampus, PD proton density, rCBF regional cerebral blood flow, ROI region-of-interest.

Structural scans were preprocessed using the N3 algorithm [60]. Hippocampus/subfield masks were generated for each participant from their preprocessed structural T1 scan by using the MAGeT Brain (multiple automatically generated templates of different brains) toolbox [61–63] (Fig. 1). This toolbox has been validated to generate hippocampus and subfield segmentations in Alzheimer’s disease and psychosis cohorts, with greater accuracy than other available toolboxes including Freesurfer 7 and FSL FIRST [62]. This is largely due to an intermediate template step which allows incorporating the neuroanatomical variability of the dataset into the segmentation of each participant, reducing registration and resampling errors, thereby yielding more accurate results. Hippocampal subfields CA2/3 were not included due to the limitations in reliably sampling these smaller regions within the spatial resolution and low signal-to-noise ratio of ASL [64]. Total GM masks were made through binarizing GM segmentations.

#### ASL sampling

Masks were registered and resampled to the individuals respective CBF map using ANTs/2.5.0 (https://github.com/ANTsX/ANTs), and the mean rCBF value was extracted per region-of-interest (ROI) per hemisphere in native space using minc-toolkit-v2/1.9.18 tools (https://bic-mni.github.io/; Fig. 1; Supplementary Methods).

### Region-of-interest (ROI) analysis

All ROI analyses were completed in R 4.2.2 (https://www.r-project.org/). Individual models assessed the effect of group (CHR-P placebo/diazepam vs. HC) or condition (CHR-P diazepam vs. placebo) on rCBF per ROI (total GM, whole hippocampus, CA1, subiculum, CA4/DG). Each model included rCBF values per hemisphere, on the basis that bilateral sampling of the same region in the same subject reflects a repeated measurement. Therefore, a group/condition*hemisphere term was included to investigate whether the effect of group or condition on rCBF significantly differed within a region based on hemisphere. Significance was set at pFDR < 0.05, corrected for multiple comparisons [65].

#### CHR-P placebo/diazepam vs. HC

Two-way mixed analysis of covariance models (ANCOVA; package stats/3.6.2) assessed rCBF differences in CHR-P placebo/diazepam compared to HC, covarying for age, sex, and global CBF. Supplementary analyses covaried for demographic characteristics which differed between groups (IQ, ethnicity) or known to affect rCBF (daily current cigarette use [66] and ROI GM volume [67]).

#### CHR-P diazepam vs. CHR-P placebo

Diazepam-induced changes in rCBF compared to placebo were assessed using linear mixed-effects models (package lme4/1.1-34) with participant ID as a random effect (intercept). Given the within-subject design and widespread expression of GABA_A_ receptors with benzodiazepine binding sites across the brain [49], global CBF was not included as a covariate. Supplementary analyses investigated potential confounding effects of global CBF, age, sex, order of scan conditions, and number of days between scans on the results.

### Exploratory/supplementary analyses

#### Voxel-wise grey matter rCBF analysis

For completeness, we explored the effects of diazepam vs. placebo on voxel-wise GM rCBF (pFDR < 0.05). The two-level modelbuild toolkit (github.com/CoBrALab/optimized_ants MultivariateTemplateConstruction) was used to generate a study-specific anatomical template (Fig. 1; Supplementary Methods). CBF maps were resampled into common-space and smoothed with a 6-mm FWHM Gaussian kernel. A voxel-wise linear mixed-effects model (R-3.5.1, RMINC-1.5.2.2, lme4 1.1–21) was used to investigate the effect of condition (CHR-P diazepam vs. placebo) on rCBF, with participant ID as random effect and masked using a study-averaged GM mask. The above procedure was repeated for investigating voxel-wise group differences in rCBF between CHR-P placebo and HC, using the one-level model template build and running a voxel-wise linear model, covarying for global CBF, age, and sex.

#### Baseline clinical characteristics and hippocampal rCBF change

Supplementary Pearson’s correlation analyses assessed whether baseline clinical characteristics (positive, negative, cognitive, anxiety, and depression symptom severity and social and role functioning) were associated with mean percent change in bilateral hippocampal rCBF under diazepam vs. placebo (see Supplementary Methods for further details on composition of clinical scores). Confounding effects of global CBF on these results were explored using partial Pearson’s correlations. Outlier detection was performed on significant correlations using Cook’s distance. Significance was set at *p* < 0.05, and multiple comparison corrections were not performed as these analyses were exploratory.

## Results

Participant demographic and clinical characteristics are detailed in Table 1. HC individuals had a significantly higher IQ and differed in terms of ethnicity compared to CHR-P, driven by an above-average mean IQ [68] and a high proportion of white ethnicity in the HC group. There were no significant differences in change between pre- and post-scan Bodily Symptom Scale [69] scores between the placebo and diazepam conditions (Supplementary Table 1).

**Table 1:** Demographic and clinical characteristics.

	CHR-P (*n* = 24)	HC (*n* = 22)	*t*/χ^2^	*p*
Age (years; mean ±SD)	24.1 ± 4.8	26.5 ± 5.1	1.7	0.09
Sex (male/female; *n*)	9/15	11/11	0.4	0.49
Ethnicity (*n*)			13.9	**0.007**
White	11	16	–	–
Asian	2	6	–	–
Black	6	0	–	–
Mixed or multiple	4	0	–	–
Other	1	0	–	–
IQ (WAIS-III short version [68]; mean ±SD)	97.6 ± 21.6	119.3 ± 16.7	4.6	**< 0.001**
Current daily cigarette use, *n* (%)	8 (33)	2 (9)	3.7	0.055
Current alcohol use, *n* (%)	18 (75)	19 (90)	1.8	0.177
Current cannabis use, *n* (%)	7 (29)	4 (19)	0.6	0.431
CAARMS [52] score (mean ±SD)				
Positive symptoms	46.4 ± 12.9	NA	–	–
Negative symptoms (*n* = 21)	29.1 ± 24.7	NA	–	–
Total (*n* = 21)	75.9 ± 29.9	NA	–	–
Global functioning score [54] (mean ± SD)				
Social	6.4 ± 1.5	NA	–	–
Role	6.1 ± 1.8	NA	–	–
Hamilton scale score (mean ± SD)				
Anxiety [55] (*n* = 22)	17.1 ± 8.5	NA	–	–
Depression [56] (*n* = 21)	13.9 ± 6.9	NA	–	–
Current antidepressant medication, *n* (%)	9	NA	–	–
Current or prior antipsychotic medication, *n* (%)	0	NA	–	–
Current benzodiazepine/hypnotic medication, *n* (%)	0	NA	–	–

*p* statistics which are < 0.05 are in bold and this denotes a significant difference between groups.

*CAARMS* comprehensive assessment of at-risk mental states, *CHR-P* clinical high-risk for psychosis, *HC* healthy control, *IQ* intelligent quotient, *WAIS* Weschler adult intelligence scale.

### ROI analysis

#### CHR-P placebo vs. HC

##### Global CBF

**Fig. 2:**
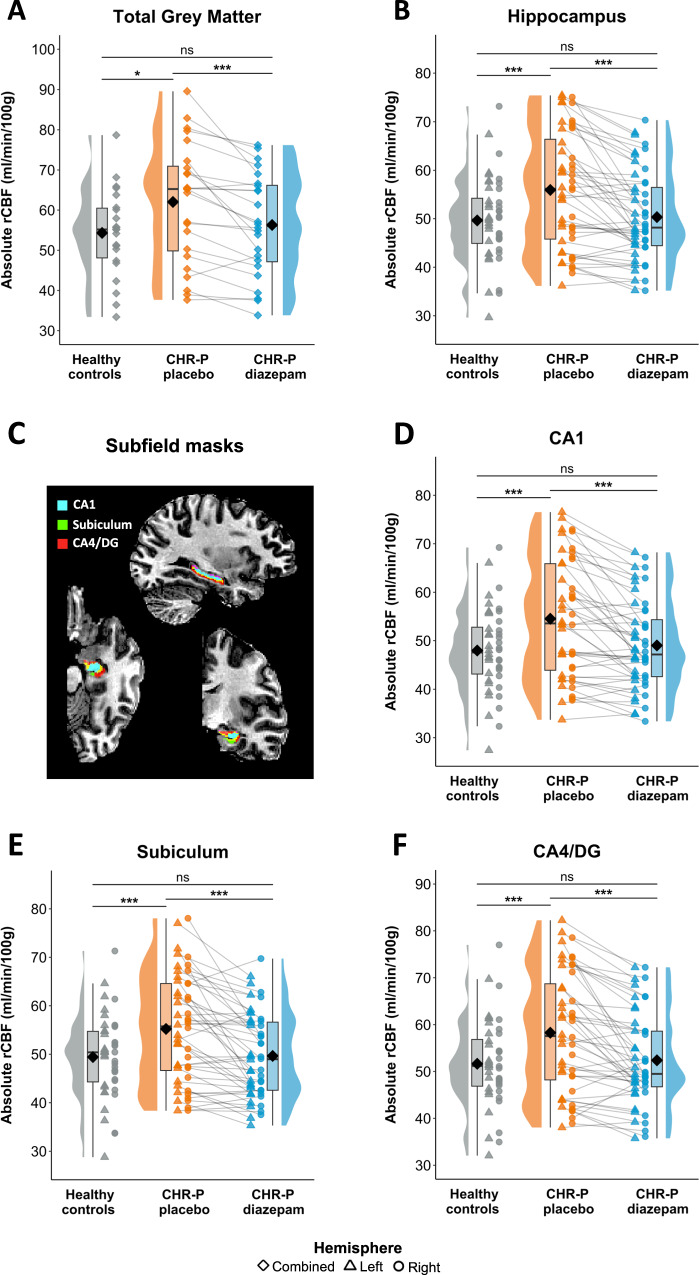
Region-of-interest rCBF findings. Absolute rCBF for HC and CHR-P participants (under placebo and diazepam) for **A** total grey matter, **B** hippocampus, and **C** hippocampus subfields, including **D** CA1, **E** subiculum, and **F** CA4/DG. CHR-P clinical high risk for psychosis, DG dentate gyrus, rCBF regional cerebral blood flow, ns non-significant; * < 0.05; *** < 0.001.

Mean CBF in total brain GM (ml/100 g/min) was significantly higher (*F*(1,42) = 5.2, *p*_FDR_ = 0.014, Cohen’s *d* = 0.59) in CHR-P individuals under placebo (62.1 ± 14.9) compared to HC (54.3 ± 10.9; Fig. 2A).

##### Hippocampus and subfield rCBF

After controlling for global CBF, age, and sex, CHR-P participants in the placebo condition had significantly higher rCBF compared to HC in the hippocampus (*F*(1,41) = 24.7, *p*_FDR_ < 0.001, Cohen’s *d* = 0.60), which did not differ between hemispheres (*F*(1,44) = 1.6, *p*_FDR_ = 0.217; Fig. 2B). Similar results were found for all subfields: **CA1** (group: *F*(1,41) = 25.8, *p*_FDR_ < 0.001, Cohen’s *d* = 0.62; group*hemisphere: *F*(1,44) = 1.1, *p*_FDR_ = 0.364; Fig. 2D), **subiculum** (group: *F*(1,41) = 25.7, *p*_FDR_ < 0.001, Cohen’s *d* = 0.59; group*hemisphere: *F*(1,44) = 1.2, *p*_FDR_ = 0.274; Fig. 2E), and **CA4/DG** (group: *F*(1,41) = 20.4, *p*_FDR_ < 0.001, Cohen’s *d* = 0.59; group*hemisphere: *F*(1,44) = 3.2, *p* = 0.082; Fig. 2F). These results did not change when adding covariates of no-interest (IQ, ethnicity, current daily cigarette use, ROI GM volume; Supplementary Table 2).

#### CHR-P diazepam vs. CHR-P placebo

##### Global CBF

In CHR-P participants, mean CBF in total brain GM was significantly lower (*t*(23) = −4.3, *p*_FDR_ < 0.001, Cohen’s *d* = −0.88) under diazepam (56.3 ± 12.7) compared to placebo (62.1 ± 14.9; Fig. 2A).

##### Hippocampus and subfield rCBF

Diazepam significantly reduced rCBF in the hippocampus (*t*(69) = −5.1, *p*_FDR_ < 0.001, Cohen’s *d* = −0.83), which did not differ between hemispheres (*t*(69) = 0.9, *p*_FDR_ = 0.366; Fig. 2B). This effect was observed across all subfields: **CA1** (condition: *t*(69) = −5.1, *p*_FDR_ < 0.001, Cohen’s *d* = −0.83; condition*hemisphere: *t*(69) = 0.8, *p*_FDR_ = 0.403; Fig. 2D), **subiculum** (condition: *t*(69) = −4.9, *p*_FDR_ < 0.001, Cohen’s *d* = −0.76; condition*hemisphere: *t*(69) = 1.1, *p*_FDR_ = 0.303; Fig. 2E), and **CA4/DG** (condition: *t*(69) = −4.7, *p*_FDR_ < 0.001, Cohen’s *d* = −0.79; condition*hemisphere: *t*(69) = 0.8, *p* = 0.405; Fig. 2F). These results did not change after controlling for global CBF, age, sex, order of scan conditions, or number of days between scans (Supplementary Table 2).

#### CHR-P diazepam vs. HC

##### Global CBF

There was no significant difference (*F*(1,42) = 0.5, *p*_FDR_ = 0.209, Cohen’s *d* = 0.17) in mean total brain GM CBF in CHR-P in the diazepam condition (56.3 ± 12.7) compared to HC (54.3 ± 10.9; Fig. 2A).

##### Hippocampus and subfield rCBF

There was no significant difference in rCBF between CHR-P under diazepam compared to HC in the hippocampus (*F*(1,41) = 0.4, *p*_FDR_ = 0.204, Cohen’s *d* = 0.08; Fig. 2B), CA1 (*F*(1,41) = 0.9, *p*_FDR_ = 0.153, Cohen’s *d* = 0.12; Fig. 2D), subiculum (*F*(1,41) = 0.8, *p*_FDR_ = 0.272, Cohen’s *d* = 0.03; Fig. 2E), or CA4/DG (*F*(1,41) = 0.3, *p*_FDR_ = 0.201; Fig. 2F, Cohen’s *d* = 0.08).

### Exploratory/supplementary analyses

#### Voxel-wise grey matter rCBF analysis

##### CHR-P placebo vs. HC

Several cortical regions showed higher (e.g., inferior/dorsolateral frontal gyrus and temporal pole) and lower (e.g., inferior parietal and middle occipital gyrus) rCBF in the CHR-P placebo condition compared to HC (p_FDR_< 0.05; Supplementary Fig. 2; Supplementary Table 3).

##### CHR-P diazepam vs. CHR-P placebo

**Fig. 3:**
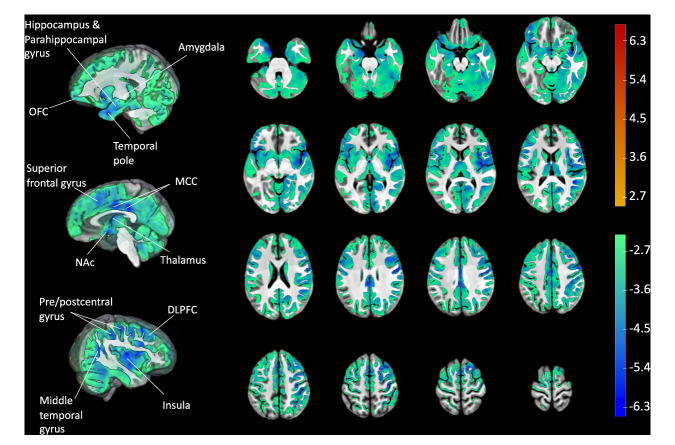
Voxel-wise grey matter rCBF findings. *T*-statistic map of drug condition (diazepam vs. placebo) effect on grey matter rCBF at the whole-brain level in CHR-P individuals from voxel-wise linear mixed effects models, thresholded and displayed at 5% FDR. Peak regions with *t*-statistic > 5 have been labeled. Color bars denote *t*-statistics which reflect 5% FDR threshold (i.e., ±2.498) and less (i.e., up to ±6.68) for both contrasts (diazepam < placebo in blue/green and placebo < diazepam in yellow/red). N.B. there were no significant voxels at 5% FDR threshold for placebo < diazepam. DLPFC dorsolateral prefrontal cortex, MCC middle cingulate cortex, NAc nucleus accumbens, OFC orbitofrontal cortex, rCBF regional cerebral blood flow.

There was a global pattern of reduced rCBF under diazepam vs. placebo (Fig. 3; Supplementary Table 4). Peak voxels (all p_FDR_ < 0.01) were located in temporal (temporal pole, parahippocampal gyrus, hippocampus, amygdala, middle temporal gyrus), parietal (pre/post central gyrus, middle cingulate), frontal (dorsolateral prefrontal cortex, ventromedial orbitofrontal cortex, insula, superior frontal gyrus), and occipital (lingual gyrus, occipital gyrus) regions, cerebellum, and subcortical regions (thalamus, putamen, caudate, and nucleus accumbens).

#### Clinical characteristics and hippocampal rCBF change

**Fig. 4:**
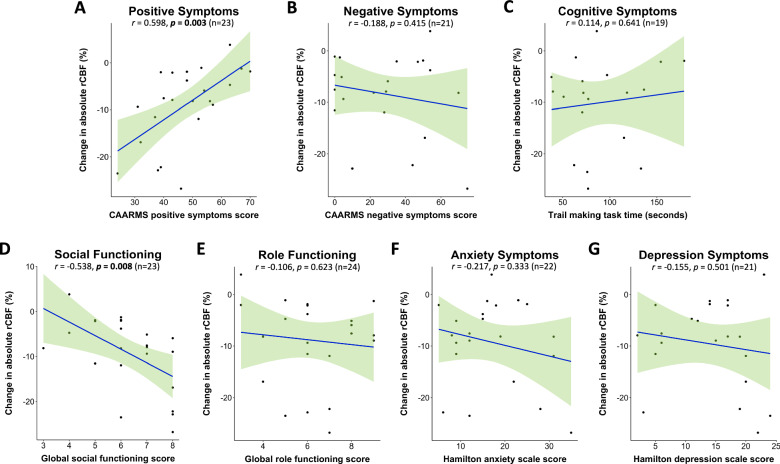
Association between baseline clinical characteristics and diazepam-induced hippocampal rCBF changes. Pearson correlations between change in absolute hippocampal rCBF by diazepam vs. placebo and baseline clinical characteristics (at assessment visit): **A** positive symptoms (*n* = 23), **B** negative symptoms (*n* = 21), **C** cognitive functioning (*n* = 19), **D** social functioning (*n* = 23), **E** role functioning (*n* = 24), **F** anxiety symptoms (*n* = 22), and **G** depression symptoms (*n* = 21). N.B. for panels D & E a higher score denotes *less* impairment, whilst for all other panels a higher score/time denotes *higher* symptom severity. Shaded light green areas reflect 95% confidence intervals. The number of participants differs between panels due to the removal of outliers or missing data. rCBF regional cerebral blood flow.

Exploratory analyses revealed significant associations between baseline clinical characteristics and change in hippocampal rCBF, such that higher baseline positive symptom severity (*r* = 0.494, *p* = 0.014) and poorer social functioning (*r* = −0.416, *p* = 0.043) correlated with less reduction in hippocampal rCBF following diazepam vs. placebo. Removal of outliers strengthened these correlations (positive symptoms: *r* = 0.598, *p* = 0.003; Fig. 4A; social functioning: *r* = −0.538, *p* = 0.008; Fig 4D). There were no significant correlations between diazepam-induced change in whole hippocampal rCBF and baseline negative (*r* = −0.188, *p* = 0.415; Fig. 4B), cognitive (*r* = 0.114, *p* = 0.641; Fig. 4C), anxiety (*r* = −0.217, *p* = 0.333; Fig. 4F), or depression (*r* = −0.155, *p* = 0.501; Fig. 4G) symptoms or role functioning (*r* = −0.106, *p* = 0.623; Fig. 4E). Most of these results remained unchanged when adding global CBF as covariate (Supplementary Table 5). There were no significant associations between baseline symptoms/functioning and hippocampal rCBF under placebo (Supplementary Table 6).

## Discussion

In our study, CHR-P individuals under placebo showed significantly higher hippocampal rCBF compared to HC. Following diazepam challenge, hippocampal rCBF in CHR-P individuals was significantly reduced compared to placebo and normalized to HC levels. This effect was also evident across all hippocampal subfields. These results are consistent with data from psychosis-relevant preclinical models and lend new empirical support for the development and investigation of more hippocampal-selective GABA-enhancing compounds as a potential therapeutic strategy in early psychosis.

Our finding of elevated hippocampal rCBF in CHR-P (under placebo) compared to HC is consistent with previous studies in CHR-P individuals [38–42]. Although we also identified higher global CBF, as found previously [40], hippocampal rCBF remained significantly elevated after controlling for global CBF, suggesting this region is particularly hyperactive. However, it is important to note we did not observe elevated hippocampal rCBF at the whole-brain level. Hippocampal rCBF was still significantly higher in CHR-P (under placebo) vs. HC on the ROI level after controlling for GM volume within that region, suggesting it was not driven by putative underlying GM differences in CHR-P individuals [70]. We hypothesized that elevations would be most pronounced in the CA1 subfield, based on prior rCBV studies in CHR-P individuals [38, 39, 42] and current models of psychosis pathophysiology [7]. However, in the ROI analysis, we observed similarly elevated rCBF across CA1, subiculum, and CA4/DG. Our finding may be related to the reduced spatial resolution of ASL compared to gadolinium-contrasted MRI as used in prior studies [38, 39, 42]. In addition, we sampled rCBF across the whole length of each subfield, instead of only anterior portions as done in those previous studies [38, 39, 42], hence it is possible that posterior portions also showed hyperactivity. Indeed, prior ASL studies in CHR-P [40, 41] and high schizotypy [53] individuals reported peak increases within the body/tail of the hippocampus. Additionally, our study used rCBF which is more tightly coupled to neuronal activity than rCBV [71], sampled in native vs. common space thereby circumventing normalization errors [72], and sampled with individual hippocampal/subfield masks thereby affording higher accuracy given the neuroanatomical diversity among individuals [73].

In line with our second hypothesis, diazepam significantly reduced hippocampal and subfield rCBF vs. placebo in CHR-P individuals, to the extent that rCBF was no longer significantly different to HC. This finding aligns with predictions from preclinical studies [34]. In the MAM rodent model, hippocampal hyperactivity is associated with a local reduction of GABAergic PV+ interneurons [28–30], and increasing GABAergic inhibition by hippocampal infusion of an α5-GABA_A_ PAM normalizes hippocampal hyperactivity [34]. Importantly, repeated oral administration of diazepam in peripubertal MAM rats prevents local PV+ interneuron loss [36] and the emergence of a hyperdopaminergic state at adulthood [35]. A likely mechanism is downregulation of amygdala-hippocampal overdrive, which causes PV+ interneuron loss in the hippocampus and, consequently, hippocampal hyperactivity. This is supported by findings that direct hippocampal infusion of the benzodiazepine midazolam normalizes increased dopamine neuron firing in the VTA of adult MAM rats [33]. Our finding of diazepam-induced reductions in rCBF across all hippocampal subfields aligns with the pharmacology of benzodiazepines and GABA_A_ receptor distribution [50, 74]. Benzodiazepines are PAMs of the GABA_A_ receptor via the benzodiazepine site, composed of an α1-3/α5 and gamma subunit [75]. Whilst GABA_A_ receptors are expressed on several cell types and sites [76], most commonly benzodiazepine binding facilitates greater hyperpolarization of post-synaptic glutamatergic pyramidal cells and reduced pyramidal cell firing [75], resulting in reduced metabolic requirements and, therefore, reduced rCBF [77]. α1-3/α5-GABA_A_ receptors (and therefore benzodiazepine sites) are highly expressed across the hippocampus [49, 50], and although there are slight differences in the levels of α1-3/α5 receptor subunits across hippocampal subfields [74], hippocampal subfields are highly interconnected [75]. Therefore, it is intuitive that diazepam was associated with a similar magnitude of reduction in rCBF across subfields.

Complementary voxel-wise analyses revealed rCBF reductions across multiple other cortical and subcortical regions in the diazepam condition. The largest reductions were seen in the pre/post central gyrus and inferior frontal regions, areas with the highest benzodiazepine receptor binding sites [49] and which receive projections from the hippocampus [18, 19]. Large rCBF reductions were also seen in the striatum, ventromedial PFC, and amygdala, which together with the hippocampus compose a cortico-limbic-striatal circuit proposed to be central to the pathophysiology of psychosis [22]. Whilst the hippocampus projects directly to these regions [78–80], it is not possible to determine whether reductions in rCBF here are due to primary drug effects (i.e., due to local increases in GABAergic inhibition), or secondary, downstream effects deriving from drug-induced reductions in hippocampal hyperactivity. The reductions in rCBF observed in further cortical regions may be related to the ubiquitous binding profile of benzodiazepines. α1–α3 receptor subunits, implicated in benzodiazepine-related side effects such as sedation and addiction, show widespread cortical distribution [81]. Conversely, α5-GABA_A_ receptors are preferentially expressed in the hippocampus [50, 74], and are not associated with such side effects [81]. PET studies have demonstrated that compared to healthy controls, antipsychotic-free patients with schizophrenia showed reduced hippocampal binding of an α5-GABA_A_ selective ligand [82], but along with CHR-P individuals [83], show no differences with less specific α1-3,5-GABA_A_ ligands [84–87]. In line with this, evidence from the MAM model shows (i) α5 but not α1-3-GABA_A_ receptors are reduced in the subiculum and CA1 [88], (ii) overexpression of the α5, but not α1, subunit normalizes hippocampal hyperactivity [32], and (iii) an α5-GABA_A_ PAM was able to normalize hippocampal hyperactivity [34] and attenuate VTA dopaminergic firing to a greater extent than the non-specific benzodiazepine midazolam [33]. Taken together, pharmacological agents with high selectivity for α5-GABA_A_ receptors may be able to regulate hippocampal hyperactivity more specifically in psychosis while potentially avoiding some of the unwanted side effects of less-selective benzodiazepines. While several α5-GABA_A_ PAMs exist [89, 90], none have yet proceeded to clinical development for psychosis.

Finally, exploratory analyses suggested that diazepam-induced reductions in hippocampal rCBF were smallest in CHR-P participants with higher baseline positive symptoms severity and poorer social functioning, although no significant associations were observed between symptoms/functioning and hippocampal rCBF under placebo. Interpretation of these findings is limited by low power for examining correlations between clinical and imaging variables, and differences in timing between clinical and MRI measurements (i.e., 1st and 2nd scan were ~2 and ~6 weeks after assessment visit). Nonetheless, current theories propose persistent hyperactivity (i.e., excessive glutamate release) in the hippocampus of CHR-P individuals may lead to atrophy of neuropil and PV+ inhibitory interneurons [7]. Therefore, diazepam may not be able to downregulate rCBF as effectively in CHR-P individuals with a more severe clinical profile and with a potentially greater degree of GABAergic dysfunction. Following development of a primary psychotic disorder, rCBF alterations appear to change with disorder progression and antipsychotic treatment. Whilst higher temporal lobe rCBF has also been reported in drug-naïve first episode psychosis individuals [91], no significant temporal rCBF differences were found in individuals with prior [92] or current [93, 94] antipsychotic-treatment, and people with chronic schizophrenia show temporal hypoperfusion [95]. In this regard, preclinical work in MAM-treated rats revealed that antipsychotic exposure blocked the therapeutic effects of GABA-enhancing compounds on psychosis-relevant phenotypes [96]. Taken together, this evidence suggests that, for individuals with a primary psychotic disorder, GABA-enhancing compounds may only be efficacious for regulating hippocampal hyperactivity when administered prior to initiating antipsychotic treatment.

Overall, this experimental medicine study presents first-in-human evidence of successful down-regulation of hippocampal hyperactivity by pharmacological modulation of the GABAergic system in CHR-P individuals. We used a robust gold-standard study design, with an adequately powered sample based on prior methodological research [97] and retrospective power analysis: with an effect size range of Cohen’s *d* = 0.76–0.88 and sample size of *n* = 24 we had an achieved power of 97–99%. All participants in our study were antipsychotic-naïve, avoiding the known effects of antipsychotics on rCBF or the GABAergic system [96, 98, 99]. Subjective effects related to sleep/sedation did not differ between the placebo and diazepam conditions, indicating that these were unlikely to affect the observed rCBF differences between conditions. We used advanced computational neuroimaging methods to segment the hippocampal subfields with high accuracy and maintained this level of accuracy by sampling rCBF with participant-specific masks in native space. We used ASL, a highly suitable neuroimaging measure for investigating the effects of a GABAergic drug challenge on hippocampal function, given it measures rCBF in a fully quantitative, non-invasive manner.

This study also has some limitations. Firstly, despite segmenting all hippocampal subfields, we could not reliably sample the smaller CA2/3 subfield due to the spatial resolution of ASL. Additionally, we were not able to restrict sampling of the hippocampal subfields to only the anterior sections (which are relevant in the pathophysiology of psychosis [7]) due to limitations of the segmentation methodology. Secondly, unlike the CHR-P group, the HC group were scanned in the absence of a placebo condition, which may have impacted rCBF. Thirdly, our study sample was smaller than previous studies comparing hippocampal rCBF between HC and CHR-P [40, 41], hence our analysis comparing HC and CHR-P individuals under the diazepam condition may have been underpowered to detect a significant difference. Finally, we did not include data on specific clinical outcomes (such as transition to psychosis) because the study was not designed nor powered for this.

## Conclusions

This study provides first evidence that a single dose of a non-specific GABA-enhancing drug like diazepam can significantly reduce hippocampal and subfield hyperactivity in CHR-P individuals and normalize it to HC levels. Diazepam-associated reductions in rCBF were also observed in other cortico-limbic-striatal regions, supporting further network-based investigations of whether diazepam can modulate this circuit in CHR-P individuals. Furthermore, the results validate the use of ASL and native-space hippocampal and subfield sampling as viable biomarker endpoints for the development of more hippocampal-selective GABA-enhancing compounds for psychosis prevention.

### Supplementary information

Supplemental Material

## Data Availability

Data will be made freely available upon publication (10.6084/m9.figshare.24763839), including (i) mean rCBF values per subject per ROI per hemisphere per condition, and (ii) coding scripts for the MRI preprocessing pipeline (run in Unix/shell) and generation of figures (run in R).
